# RELATIONSHIP OF PREOPERATIVE COMPREHENSIVE GERIATRIC ASSESSMENT TO HEALTH STATUS IN OLDER ADULTS

**DOI:** 10.1093/geroni/igz038.2525

**Published:** 2019-11-08

**Authors:** Ji Yeon Lee, Kwang Joon Kim, Chang Oh Kim, Kyung Hee Lee

**Affiliations:** Yonsei University, Seoul, Korea, Republic of

## Abstract

Although comprehensive geriatric assessment has been widely used in surgical older adults, its relationship to health status has not been fully identified. This study aimed to examine the relationships of preoperative comprehensive geriatric assessment to frailty and length of stay. This was a descriptive study based on multi-professional health assessments found in electronic medical records. Study participants were 150 older adults in a neurosurgical department. The comprehensive geriatric assessment was comprised of nutrition, functional status, physical activity, depression, cognition, and basic items such as the Timed Up and Go test, grip strength, and self-rated health. Frailty level and length of stay were dependent variables which represented health status. The result showed that instrumental activities of daily living, physical activity, nutrition, self-rated health, and cognition were significantly associated with frailty. Specifically, comparing robustness with pre-frail and frail level, worseness in the instrumental activities of daily living, self-rated health, physical activity, and nutrition were associated with frailty. With progression of frailty level from pre-frail to frail, the worse score in the cognitive function and self-rated health were associated with frailty. In addition, more depressive symptoms, postoperative complications, and prolonged in the Timed Up and Go test were associated with lengthened hospital stay. Older adults with worsened status in physical, emotional, or cognitive function tended to be frail and stay longer in the hospital. Clinicians need to pay attention to the subcomponents of the comprehensive geriatric assessment and are encouraged to implement it to improve health status of surgical older adults.

